# Development of a tic service model for children and young people in England: a Delphi study

**DOI:** 10.1136/bmjopen-2025-107534

**Published:** 2026-04-28

**Authors:** Sophie S Hall, Nikita R Rattu, Charlotte L Hall, Joseph Kilgariff, Tara Murphy, Nadya James, Emma McNally, Suzanne Rimmer, Louise Thomson, Jaxon H Kramer, Madeleine J Groom

**Affiliations:** 1 Nottingham Clinical Trials Unit, University of Nottingham School of Medicine, Nottingham, UK; 2 Academic Unit of Mental Health & Clinical Neuroscience, University of Nottingham School of Medicine, Nottingham, UK; 3 Nottinghamshire Healthcare NHS Foundation Trust, Nottingham, UK; 4 NIHR MindTech MedTech Health Research Centre, Institute of Mental Health, University of Nottingham, Nottingham, UK; 5 NIHR Nottingham Biomedical Research Centre, Institute of Mental Health, University of Nottingham School of Medicine, Nottingham, UK; 6 University College London Great Ormond Street Institute of Child Health, London, UK; 7 Great Ormond Street Hospital for Children NHS Foundation Trust, London, UK; 8 Nottingham University Hospitals NHS Trust, Nottingham, UK; 9 Tourettes Action, Farnborough, UK; 10 Formerly NHS Healthcare Commissioner, Now Retired, Merseyside, UK; 11 Wandsworth Tier 3 CAMHS, South West London & St. George’s NHS Trust, London, UK

**Keywords:** MENTAL HEALTH, Health Services, Child & adolescent psychiatry

## Abstract

**Abstract:**

**Objectives:**

To develop an evidence-based, consensus-driven service model for the identification, assessment and treatment of tic disorders in children and young people (CYP) in England, addressing the absence of dedicated pathways and national clinical guidance.

**Design:**

Two-stage consensus study comprising a Delphi survey, expert/patient and public involvement (PPI) review and regional stakeholder workshops.

**Setting:**

UK healthcare and community settings relevant to tic disorder assessment and management (primary care, neurodevelopmental services, child and adolescent mental health services).

**Participants:**

Stage 1: UK-based clinicians, researchers and practitioners with expertise in tic disorders (Delphi panel; n=49; 98% retention across rounds). Stage 2: regional stakeholders including clinicians, commissioners, service managers, third-sector representatives and parents/carers (n=36). Eligibility required relevant professional or lived experience; no exclusions applied beyond this criterion.

**Primary outcome:**

Identification of a consensus-based component of a best-practice service model for tic disorders and barriers and facilitators to implementation across regional pathways.

**Results:**

The Delphi process generated consensus on 40 core components, refined to 43 following expert and PPI review. Agreed features included referral criteria, comprehensive assessment, psychoeducation, behavioural interventions, pharmacological options and integrated cross-service working. Stakeholder workshops highlighted key implementation challenges, including workforce training, funding constraints and coordination across neurodevelopmental and mental health services, informing practical adaptations to the model.

**Conclusions:**

This consensus-informed service model provides structured, UK-specific guidance to support earlier identification, appropriate intervention and improved care coordination for CYP with tic disorders. Future research should assess real-world implementation and impact.

**Clinical implications:**

The model offers actionable recommendations for referral pathways, intervention provision and service configuration. Adoption may reduce diagnostic delays, minimise misdiagnosis and strengthen collaboration between primary, neurodevelopmental and mental health services, leading to improved outcomes for CYP with tic disorders.

**STRENGTHS AND LIMITATIONS OF THIS STUDY**

- This study used a rigorous two-stage consensus design, combining a three-round Delphi survey with subsequent expert discussion to agree the final service model components.
- Targeted recruitment ensured representation from clinical, academic and commissioning experts, although Delphi participants were predominantly white and UK-based.
- Retention across Delphi rounds was high, strengthening reliability of consensus.
- Regional stakeholder workshops enabled triangulation of findings and identification of contextual implementation barriers and facilitators, although discussions were not audio-recorded and relied on structured note-taking.
- Patient and public involvement informed item refinement and pathway wording but lived-experience representatives were not included in the Delphi survey itself due to technical complexity of survey items.

## Background

 Tic disorders, including Tourette syndrome (TS), impact approximately 1% of children and young people (CYP), equating to around 110 000 individuals in England.^1 2^ Tics are sudden, repetitive, involuntary movements or vocalisations such as eye rolling, head jerking or throat clearing. These symptoms often appear in early childhood, typically between ages 3 and 8 years and may persist throughout life for many individuals.^3^ Tic disorders have approximately the same prevalence as autism and epilepsy but unlike those conditions, there are no clear service pathways and no guidance from independent public bodies, including National Institute for Health and Care Excellence (NICE) in a UK context. Furthermore, NICE guidelines state that tics are likely to remit and only warrant a referral to specialist services if there is a co-occurring mental health or neurodevelopmental condition.^4^ However, recently published evidence confirms that tics persist for the majority of CYP with a tic disorder and cause psychological and/or functional impairment for many.^5^ Our recent research confirms that there are very few service pathways offering diagnostic assessment, treatment and management of tics in England.^6^ CYP are often referred to inappropriate services that are ill-equipped to manage tics, leading to inefficiencies; ensuring access to services that can provide diagnostic assessment and management of tics can reduce this waste and result in cost savings.^7^

For CYP with tic disorders, timely access to specialists with the expertise to evaluate and accurately diagnose tics is critical. Over time, tics can intensify and become more complex, involving sequences of movements—such as sudden leg kicks or full-body contortions—or verbal utterances that may be complete phrases or sentences. These symptoms can be alarming for patients and their families who do not have clarity about a diagnosis of treatment. CYP with TS also face elevated risks for depression, anxiety and suicide in adulthood,^8^ as well as social stigma and reduced academic success.^9^ Additionally, many people experience physical pain, injury or disability from tics.^10^ Psychiatric comorbidities are prevalent among individuals with TS, with research indicating that 50% or more also have attention-deficit/hyperactivity disorder (ADHD) or obsessive-compulsive disorder (OCD).^11^ Additionally, around one-third are affected by anxiety or mood disorders.^11 12^ Those diagnosed with a tic disorder, particularly severe cases, require ongoing review and support. Enhancing services for tic disorders and expanding access to trained professionals skilled in tic therapies could help lower these costs and improve outcomes for CYP and their families. However, our recent study found that 79% of healthcare professionals (HCPs) (including psychiatrists, psychologists, nurses, paediatricians and neurologists) working in children’s services in England reported that they do not have sufficient training or resources to assess and treat people with tics.^6^

Despite the implications of not receiving timely diagnosis and treatment for CYP with tics, data reported by NICE reveals that a recent UK survey of 1508 people with tics/TS found that 63% of children, young people and adults waited over a year for diagnostic assessment, with 23% waiting 3 years or more. Of those who received a diagnosis, over 60% had not received any treatment for tics by the time of the survey. This aligns with other qualitative studies showing families’ difficulties in accessing care and the associated psychological distress^13 14^ and lengthy waits for diagnosis.^15^

Focus groups with families affected by tic disorders emphasised the urgent need for a clear management pathway, better-trained professionals, improved communication during referrals and prioritised treatment for related mental health issues.^14^ The European clinical guidelines for TS^16–18^ go some way to suggesting a pathway for managing tic disorders. The guidelines recommend beginning with psychoeducation to help patients, families and educators understand the condition, followed by behavioural therapies as the primary treatment approach, based on evidence that these are effective treatments.^19^ More recent guidelines for tic disorders have been developed to include the management of tics in the context of co-occurring conditions.^20 21^ However, they do not offer guidance on how to structure a service pathway for CYP with tics, nor do they clarify best practices for organising assessment and treatment within the UK healthcare context.

As part of our preliminary investigation in this area, we identified previous work that aimed to establish consensus among 10 HCPs on a UK tic service model, including the model’s ideal structure, target age range, duration of service input and recommended interventions.^22^ This served as a valuable foundation for further development. To bridge the gap between evidence and practice in the UK, there is now an urgent need to extend this in a larger sample and develop a practical, evidence-based service model supported by clear guidance. Such a model is crucial for reducing inequalities in service access and ensuring consistent, high-quality care for CYP with tics across healthcare settings.

Here, we report a two-stage process to develop a UK-based tic service model for CYP to provide a clear pathway for the referral, assessment and treatment of tic disorders. The first stage comprised a Delphi survey of experts in healthcare for tic disorders to identify the core features of a best-practice service model. The second stage was a series of stakeholder workshops with HCPs and those involved in the design and delivery of services to identify potential barriers to implementing a best-practice service model (pre-implementation) and to explore factors that may overcome these barriers using the Consolidated Framework for Implementation Research (CFIR)^23^ as an overarching framework.

## Methods

### Patient and public involvement

Patient and public involvement (PPI) was embedded throughout the study. Lived experience was defined as being a parent or carer of a child or young person with a tic disorder and having experience of accessing healthcare for their child/young person’s tics. A PPI panel (n=10) was formed after advertising nationally. The panel comprised mothers (n=8) and fathers, who were mostly white British (n=8) with two members from ethnic minority groups, recruited from 10 different regions of England. The panel was chaired by a parent of a young person with TS who also has a professional role as the CEO of the national charity, Tourettes Action (coauthor EM). The PPI panel met every few months to review the study design (including some aspects published previously^6^), share their own experience of healthcare pathways for CYP with tics and, in the later stages of the study, to refine the overall pathway structure and design. They also co-created dissemination materials to share the findings with the lived experience community.

We did not recruit parents/carers or CYP with tic disorders to participate in the Delphi consensus process because the technical language and service design terminology used across the 61 items required a high-level understanding of healthcare pathways. Instead, the PPI panel described above and lived experience representatives from charities and support groups for TS contributed to the refinement and overall structure of the pathway following the consensus workshop. Their input shaped key components of the pathway, including the referral criteria and the centrality of multidisciplinary liaison between services. Charity and support group representatives (including the PPI panel chair) attended the regional workshops.

### Stage 1: consensus approach—Delphi survey

#### Recruitment and participants

Sample size guidelines for Delphi studies vary from as low as 10 participants to up to 50,^24 25^ with other recommendations suggesting the group characteristics are taken into consideration ensuring there are approximately 5–10 participants representing different groups.^26^ As our target population consisted of experts in the field of tic disorders, including academic researchers and clinicians working with CYP, we sought to recruit a sufficiently large and diverse expert panel to capture a broad range of perspectives. Therefore, we aimed to recruit 30–50 participants to ensure the robustness of the consensus process. Participants were invited via email, starting with a list of experts identified by the study team through publicly available information (eg, clinicians known to have specialist expertise in tics and academics with peer-reviewed publications in this field). Additional participants were then recruited through snowball sampling from this initial group. A total of 125 invitations were sent. The inclusion criteria for participation were (a) a researcher who has been listed as an author in ≥3 peer-reviewed publications in the field of tic disorders including TS or (b) a professional who practices clinically in the UK and has been working clinically (with dedicated/protected clinical time allotted to work involving TS/TD populations) for ≥2 years.

#### Items

61 Delphi items were developed based on targeted reviews of the relevant literature^16–18 27 28^ and a series of online workshops with members of our Study Advisory Group, comprised of three expert researchers, three HCPs (consultant paediatric neuropsychologist, paediatrician, advanced nurse practitioner) and two representatives from charities and support groups for tic disorders in the UK. Clinical experts within the advisory group drew on previously published guidelines to inform selection and wording of items for the Delphi survey.

#### Approach

A three-round Delphi survey was hosted using Welphi, a specialist software for Delphi surveys.^29^ The first round of the Delphi Survey opened on 29 February 2024 and closed on 15 April 2024. The second round opened on 2 May 2024 and closed on 5 June 2024. The third round opened on 17 June 2024 and closed on 16 July 2024. Participants were only invited to complete the subsequent survey round if they fully completed the prior round.

**Figure 1:**
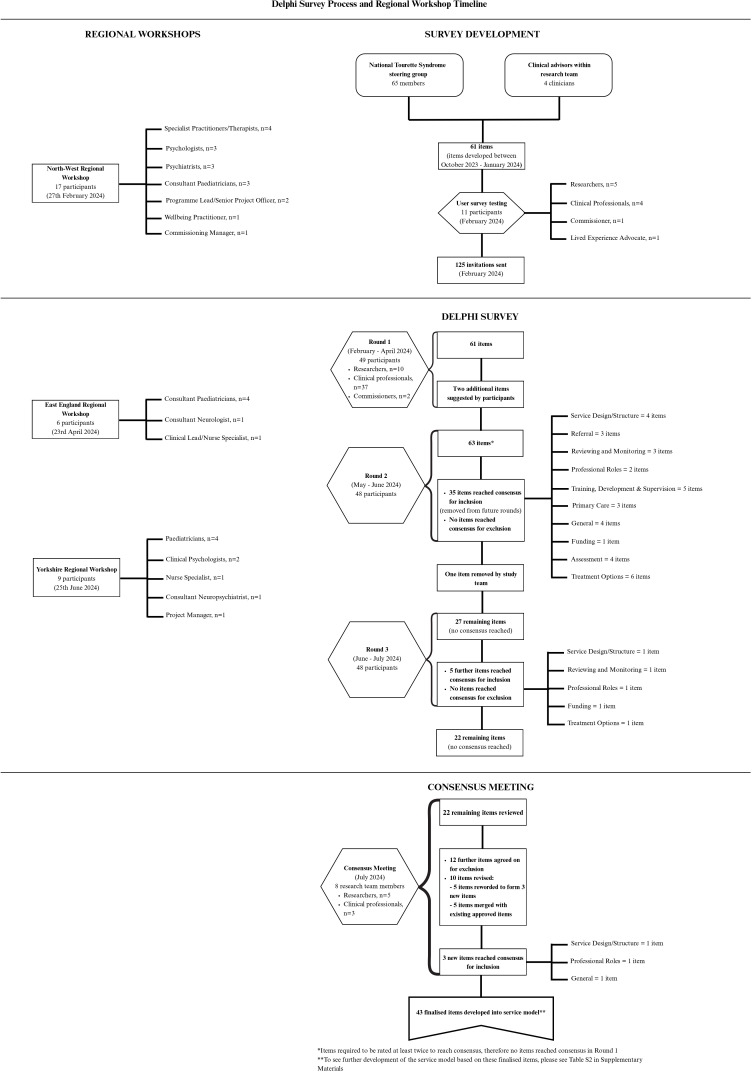
A summary of the Delphi process to reach consensus on the core items to include in a UK tic service model for children and young people.

After reading the Participant Information Sheet and providing consent, participants were given the opportunity to be entered into a prize draw to receive £50 (GBP) of online shopping vouchers if they completed all three rounds. Participants were required to complete a brief demographic questionnaire before proceeding to the 61 item Delphi survey (see online supplemental materials, table S1). Delphi responses were collected anonymously, and demographic information was stored separately from item-level ratings. This ensured participants could provide honest and independent judgements and prevented item-level analyses by stakeholder group. Delphi items were presented in categories. For example, items that related to referrals were presented on one page under the heading ‘Referrals’. The item rating process is displayed in figure 1.

Participants were asked to rate each item for its importance in a service model for CYP in the UK with tic disorders. Each item was rated using a 9-point scale (1–3: ‘Not important to include in a tic service model for CYP’; 4–6: ‘Important but not critical to include in a tic service model for CYP’; 7–9: ‘Critical to include in a tic service model for CYP’), there was also the option to choose ‘Unable to rate’.

An option to add new items was presented as part of the first survey round only. These suggestions were reviewed by the Study Advisory Group for consideration to include in round 2.

#### Analysis

Each item was tabulated and categorised as follows:

- Consensus in=≥70% participants scoring 7–9 and <15% participants scoring 1–3.
- Consensus out=≥70% participants scoring 1–3 and <15% of participants scoring 7–9.
- No consensus=anything else.

After round 2, all items which had reached consensus (in/out) were removed from the third and final survey round. Items which did not reach consensus after the third round were further discussed in a 2-hour online consensus workshop attended by eight members, including experts in tic disorders by research and clinical practice (consultant paediatric neuropsychologist, advanced clinical practitioners, neurodiversity consultants). After discussion of each item, the group voted as to whether the item should be included or excluded in the service model, using the Microsoft Poll function in Microsoft Teams. The final 43 items were also presented to our PPI panel (n=10 parents of CYP with tics), which included three re-worked items following the consensus workshop for critical evaluation. Discussions in the consensus workshop were documented using structured, outcome-focused notes rather than audio recordings, reflecting standard practice in many consensus workshops.^30^

### Stage 2: regional workshops

#### Recruitment and participants

HCPs currently working in services for CYP’s mental health, neurodevelopmental or neurological disorders from three regions of England (East of England, the Northwest and the combined Yorkshire-Humber and North Lincolnshire regions) were invited to participate in a workshop to share their experiences of current provision for tic disorders in their service. The workshops were advertised through the TS Steering Group—UK (weblink), the Health Innovation Networks (organisations responsible for supporting the implementation of research into practice in England), the charities Tourettes Action and TIC-Hull Yorkshire and further snowballing from these sources. The three workshops took place between February and June 2024.

#### Approach

The main aims of the workshops were to identify factors that would be likely to influence implementation of the expert-designed pathway for tic services in those regions. Each workshop began with a presentation by the study team and a lived experience advocate (eg, from a national or local support group for tics). These were designed to provide general information and context and to orientate participants towards the topics of group discussion following the presentations. Following the presentations, workshop participants were organised into small groups (approximately six per group) to discuss current provision for tic disorders within each region, including the components of an ideal service model, potential barriers to service change and potential solutions or factors that might mitigate those barriers. To promote open discussion, sessions were not audio-recorded. Instead, comprehensive, action-focused notes were recorded in real time by members of the research team to document key discussion points and outcomes. After all workshops had been completed, the summaries from each workshop were grouped and aligned with the CFIR.^23^ The CFIR is an evidence-based implementation framework which can be used to guide the systematic assessment of barriers and facilitators for complex health interventions. The CFIR framework consists of five domains, broad categories of influence on implementation processes (the innovation/intervention itself; the outer setting, the inner setting; the individuals; the implementation process) and more specific and detailed constructs within each domain.^28^ We used this as a guide to evaluate barriers and facilitators within these domains, as a pre-implementation assessment. The knowledge gained during the workshops helped us to word the pathway features (the final wording of the items approved through the Delphi) to enhance future acceptability/implementation in practice. The Delphi items themselves were not presented or discussed at the workshops

## Results

### Stage 1: consensus approach

49 participants completed the first survey round (39% response rate from the 125 invitations sent). Demographic characteristics of the participants are shown in table 1.

**Table 1:** Demographic characteristics of the Delphi survey participants

	Round 1 (n=49), % (n)	Rounds 2 and 3 (n=48), % (n)
Role		
Researcher	20.4 (10)	20.8 (10)
Healthcare professional working with CYP with tic disorders	75.5 (37)	75 (36)
Commissioner	4.1 (2)	4.2 (2)
Gender		
Female	69.4 (34)	68.8 (33)
Male	30.6 (15)	31.2 (15)
Ethnicity		
White	81.6 (40)	81.3 (39)
Asian/Asian British	12.2 (6)	12.5 (6)
Mixed/multiple	4.1 (2)	4.2 (2)
Other	2 (1)	2.1 (1)
Region		
East Midlands	32.7 (16)	33.3 (16)
East of England	4.1 (2)	4.2 (2)
London	24.5 (12)	22.9 (11)
Northwest	14.3 (7)	14.6 (7)
Southeast	4.1 (2)	4.2 (2)
Southwest	8.2 (4)	8.3 (4)
West Midlands	2 (1)	2.1 (1)
Yorkshire and Humber	4.1 (2)	4.2 (2)
Prefer not to say	6.1 (3)	6.3 (3)

CYP, children and young people.

Full results of each round of the Delphi survey are documented in figure 1. After reviewing two suggestions for new items provided by participants in round 1 and informed by discussions in the first two regional workshops, two items were added to the second survey round, resulting in a revised total of 63 items (see online supplemental table S1).

The second survey round yielded a 98% completion rate (n=48). From the 63 items, 35 items reached consensus to be included in a service model for CYP with tics, with no items being rejected. Agreement was not reached for 27 items and therefore these were further evaluated in round 3.

The third survey round was completed by all round 2 participants (n=48; 98% completion rate from round 1). From the 27 items rated, 5 items reached consensus to be included in the service model, with no items being rejected. Agreement was not reached for 22 items. Overall, a total of 40 items reached agreement to be included in a service model through the Delphi survey (see online supplemental table S1).

The 22 items that had not reached consensus by the end of round 3 were discussed in a consensus meeting, where attendees reviewed each item and then voted to ‘include’ or ‘exclude’ it from the service model. As part of this discussion, the attendees also considered free-text feedback provided by the Delphi participants in each round of the survey. This feedback indicated that several of these non-consensus items contained overlapping content, ambiguous wording or referenced specific professional roles rather than focusing on the underlying responsibilities of professionals delivering a tic service. During the consensus meeting, these issues were considered in detail, and 10 items were revised—either merged with existing agreed items or re-worded to improve clarity—resulting in three new consolidated items. No items were revised between survey rounds. Using the same ≥70% agreement criteria applied in the Delphi survey, all three revised items reached consensus for inclusion and another 12 items from the initial set of 22 non-consensus items were excluded, yielding a final set of 43 items.

Following the consensus meeting, the final items underwent further iterative review with the study team and PPI panel. This process resulted in additional refinements to improve clarity and applicability across different service structures. Similarly, feedback from the PPI panel highlighted the need for greater clarity in the referral criteria. While confirming a 12-month duration of tics is often required for diagnosis, families stressed that significant distress, harm or functional impairment should also trigger referral. In response, the referral criteria were revised to specify that any one of these indicators (12-month duration, distress/harm or impairment) should prompt referral.

The final tic service pathway was created from the consensus Delphi items (see online supplemental materials, table S2 for further information). Accompanying guidance was developed to provide further context and explanation, in alignment with the wording of the Delphi items. This will be made available on request to the authors.

### Stage 2: regional workshops

The three regional workshops were attended by 36 HCPs, including psychologists, neurologists, therapists, psychiatrists, paediatricians, nurse specialists, commissioners, service managers. Reoccurring topics of interest/discussion across the workshops are shown in table 2, reflecting potential barriers and related facilitators to implementing a tic service pathway. Each barrier/facilitator was aligned with the CFIR as shown in table 2 to provide a pre-implementation summary of likely barriers to implementation and how to address them. These show a range of barriers identified across CFIR domains including the intervention (innovation design and adaptability), outer setting (Financing, Policies & Laws), the inner setting (Available Resources, Relative Priority, Structural Characteristics, Compatibility, Relational Connectedness, Communications, Access to Knowledge) and individual characteristics (capability, opportunity) domains. The workshop discussions also provided the research team with insights into how to word the final items included in the service pathway.

**Table 2:** Barriers and facilitators to pathway implementation identified from the regional workshops aligned to the Consolidated Framework for Implementation Research

Potential barriers to implementation	Potential enablers of implementation	CFIR domain and constructs
Lack of funding/resources and lack of prioritisation by management	Provide evidence and information to guide and influence funding decisions and commissioning arrangements HCPs feel there is a need for change and that early intervention can reduce costs of service use later—use this as leverage to influence decision-makers Influence national policy to ensure tic service provision is mandatory (funding must therefore be allocated by ICBs)	Outer setting domain, construct F ‘Financing’ Inner setting domain, construct J ‘Available resources 1. Funding’ and construct G ‘Relative priority’
Service managers and commissioners perceive that the long waiting lists for neurodevelopmental disorders mean they are already stretched and lacking capacity and therefore cannot extend their remit to include CYP with tics within service plans	Provide evidence to decision-makers of the economic benefits (to healthcare and other social and educational systems) from implementing a tic pathway. Provide evidence of the number of potential referrals to a tic pathway.	Inner setting domain, construct J ‘Available resources’
Lack of national (NICE) guidelines for the assessment and treatment of tic disorders and lack of national safety standards and frameworks in this area undermine HCP willingness to conduct assessment and treatment of tics	Provide evidence to NICE and regulatory bodies of the need for guidelines and safety standards in this area (including the views of HCPs and lived experience support groups and charities) to support implementation of the pathway	Outer setting domain, construct E ‘Policies & Laws’
Low staff recruitment and high turnover in services for CYP mental health and neurodevelopmental disorders	Writing the tic pathway into service operating procedures will facilitate delivery through specific job roles within the service, rather than dependency on one person	Inner setting, construct A. ‘Structural characteristics 3. Work infrastructure’)
Lack of knowledge and confidence in tic assessment and treatment among staff	Provide guidelines on the provision of training and resources for staff involved in delivering the tic pathway Standardised training in assessing and treating tics for core staff working within children and young people’s mental health and neurodevelopmental services	Inner setting, construct K ‘Access to knowledge and information’ Individuals, characteristics subdomain, B. Capability
Resistance to change among HCPs; fears of ‘opening the floodgates’ (by accepting referrals for tics)	Use examples of existing tic pathways to demonstrate the economic and other benefits to the service and HCPs within the service	Individual characteristics subdomain, B. ‘Capability’ and subdomain C. ‘Opportunity’
Weak structures to support shared care and multidisciplinary working across teams	The pathway includes recommendations and guidance to support multidisciplinary team working and communication. Engage with implementation leads to tailor the pathway to local context.	Inner setting, B. ‘Relational connections’ and inner setting, C. ‘Communications’
Psychoeducation (for parents and CYP) is an essential component of a tic service pathway	Through further funding, develop a standardised, evidence-based, well-designed psychoeducation resource that can be made available to services to support pathway implementation	Innovation domain, G. ‘Innovation design’; Inner setting domain, F. ‘Compatibility’
The pathway should fit within existing service arrangements and needs to be adaptable	The pathway has been designed to be suitable for implementation in current services responsible for CYP mental health and/or neurodevelopmental disorders. Engage with implementation leads to tailor the pathway to local context (eg, approaches to screening, triage and discharge processes)	Innovation domain, D. ‘Innovation adaptability’

CFIR, Consolidated Framework for Implementation Research; CYP, children and young people; HCP, healthcare professional; ICB, Integrated Care Board; NICE, National Institute of Health and Care Excellence.

The Delphi survey and regional workshop discussion points were synchronised through iterative discussions in the study team, including clinical and PPI representatives, and evaluated with the PPI parent panel, to form the recommended service pathway and accompanying guidelines.

## Discussion

The aim of this study was to co-design (with healthcare, academic experts and PPI) a recommended service pathway for the referral, assessment, treatment and management of tics in CYP. Using a consensus-based approach we identified 43 essential features to include within the pathway. These items were then refined and developed into a flow chart and accompanying guidelines for use in mental health and paediatric services for CYP in England.

This study builds on early exploratory work in this area^22^ to develop an expert-informed service pathway for tic disorders in the UK, using an expert-consensus based approach. The essential features to include are largely congruent with the European Clinical Guidelines for TS^27^ and best practice specified in the BMJ Best Practice Guidelines.^20^ For example, expert participants in the Delphi survey confirmed the importance of using a combination of clinical interviews, physical and neurological examination and standardised tools (eg, the Yale Global Tic Severity Scale) to assess tic disorders.^18^ The Delphi survey and the Regional Workshops also identified the importance of psychoeducation as a fundamental component of tic management, while also recognising the value of Habit Reversal Therapy/Comprehensive Behavioural Intervention for Tics and Exposure Response Prevention as first-line/early-stage interventions.^28^ Remote delivery of behavioural therapy was also considered an essential feature of tic service model, which is likely to reflect the growing scientific base supporting this mode of treatment in recent years.^31^ The Delphi survey experts also recognised that behavioural approaches are not always effective or feasible for all CYP with tic disorders and therefore a tic service pathway should support the use of pharmacological treatment. Further discussion in the expert consensus meeting led to the recommendation that this can be as monotherapy or in combination with behavioural therapy.^27^

Importantly, this study also emphasised the need to understand the specific features of a tic service within the National Health Service (NHS) healthcare context in England. Several key elements were identified, including the referral process (eg, appropriate sources for referrals), systems for review and follow-up (eg, who should oversee ongoing care), the role of primary care (eg, training to identify, refer and manage shared care for tic disorders) and the necessity for appropriate commissioning and funding. Discussions in the consensus meeting endorsed qualitative comments from the Delphi survey and the regional workshop participants stipulating that the service pathway must be adaptable to fit local organisation of services, which can vary considerably across Integrated Care Boards (ICBs) in England.^6^ In particular, rather than operating as a standalone pathway, the tic service pathway must be included within current NHS healthcare provision for neurodevelopmental and/or mental health services. The choice of whether the service fits within existing neurodevelopmental/mental health services should be driven by local context and service design within specific ICBs and Places. The Delphi and regional workshop participants strongly endorsed the need for these services to liaise regularly with one another to ensure children with comorbid conditions such as ADHD, autism and OCD are supported effectively. These conditions frequently co-occur with tics,^9^ reinforcing the need for a service model which assesses and manages tics in conjunction with co-occurring symptoms, particularly when developing an appropriate treatment plan. Currently, liaison between services varies across England^6^; improving methods and frequency of communication and relational connections will be essential to the smooth integration of the expert-recommended tic service pathway presented here and will also have beneficial effects for CYP with other conditions that often sit across mental health, neurodevelopmental and paediatric services.

A key aspect of the service pathway developed here is the role of primary care practitioners in identifying tics and making a referral in accordance with the criteria specified here. These criteria encompass the chronicity of the tics (tics should be present for at least 12 months), their functional impact and their psychological/physical harm/distress. These referral criteria were written with our PPI panel and guided by the clinical and lived experience advocates in the study team. For those with lived experience, it was deemed essential that evidence of psychological/physical harm/distress or functional impairment are sufficient criteria for referral, even if the tics have been present for less than 12 months. The Delphi survey established consensus on the importance of primary care practitioners in managing shared care protocols when CYP with tics are discharged from the tic pathway but need to continue receiving medication and having routine follow-up to monitor their tics and other symptoms/difficulties. To support primary care practitioners, we are co-developing an online resource to support and enhance primary care knowledge and awareness of tics, and we will co-develop a referral process with them, building on current practice and electronic referral templates. This additional work will strengthen the delivery of these parts of the pathway.

The regional workshops with HCPs also provided valuable insights into likely barriers (and potential solutions) to implementing a tic pathway within existing services in England. This pre-implementation work was aligned with the CFIR^23^ and has provided a platform from which to design an implementation-evaluation study to implement the tic pathway in services. The three workshops were consistent in identifying barriers in the areas of commissioning and funding, lack of national clinical guidelines for tic disorders, a lack of training and expertise among HCPs working in relevant services (where a tic pathway would best ‘sit’), weak structures for shared care and multidisciplinary team working (particularly across services) and a sense of resistance to change and fears of ‘opening the floodgates’ by accepting referrals for tic disorders. These discussion points align closely to the findings from our previously published national survey of HCPs,^6^ many of whom expressed a need for more training and resources to enhance their knowledge and confidence of assessing and treating tics, and described their frustration at the current organisation of services for CYP with tics. The positivity among those attending the regional workshops also led to a number of potential solutions to these barriers, many of which are within the ‘Inner Setting’ domain of the CFIR. These are therefore ideal first targets in a future implementation-evaluation study, coupled with identifying other barriers and facilitators during the process of implementation. We are currently developing plans for further research to implement and evaluate the pathway in services in England, including how best to implement the pathway in different service structures.

The pathway is designed to fit within existing local service structures. In some areas it will sit within neurodevelopmental teams, while in others it will be delivered by CYP mental health services with paediatric or neurology input via shared care. These variations affect who leads each component rather than the pathway content, and the model accommodates this flexibility across ICBs. We are now preparing for implementation across services in England and examining how best to deliver the pathway within different service configurations.

The strengths of this study include the consensus-based methodology to identify key items for the tic service pathway and incorporating the views and preferences of PPI and HCPs throughout. Our targeted recruitment strategy for the Delphi survey ensured we had representation from researchers, clinical professionals and commissioners, providing both breadth and depth of knowledge in this area. We were able to sustain a high response rate of 98% from round 1 to round 2 and 100% from round 2 to round 3. This is higher than typical Delphi surveys where response rates in the final round are often as low as 55%–67%.^32 33^ However, our approach is not without its limitations. While the Delphi approach enables an anonymous and objective method for reaching agreement (ie, by using a pre-defined criteria for consensus), the lack of opportunity to discuss item wording means that the survey items/proposed features could be interpreted differently across the sample. However, the items were generated and refined through an iterative process of discussion with national experts in tic disorders who were able to draw on their knowledge and expertise, as well as consulting existing European and best practice guidelines.^20 27 28^ Participants in the Delphi survey did not often suggest additional items or alternative wording, indicating that the items were comprehensive and clear. Because demographic data were not linked to individual item-level ratings, it was not possible to examine voting patterns across stakeholder groups. This reflects the intentional anonymity of the Delphi design, which aims to minimise social influence and protect the independence of expert judgements. Another potential limitation is the Delphi sample composition who predominantly identified as white British. However, although only 12.5% of our final sample identified as being Asian, this exceeds the 9.3% that would be expected based on UK figures.^34^ Recruitment for the Delphi was conducted via email, beginning with experts identified through publicly available information; although many invitees were not personally known to the team and snowball sampling widened reach, this approach may still have excluded individuals less visible within established networks. A further limitation is that discussions during the consensus workshop were captured through structured, outcome-focused note-taking rather than audio recording. While this approach is widely used in consensus-building exercises, it may have resulted in the loss of some nuance in participants’ discussions. Finally, although lived experience expertise was central to the study, neither CYP with tic disorders nor their parents were included directly in the Delphi consensus survey. The technical and service configuration terminology of the Delphi items was not accessible to younger participants or those without clinical-academic expertise, and inclusion risked misinterpretation, and/or having to word the items in a way that limited their relevance to HCPs and service pathway design. To ensure lived experience informed the process in a meaningful way, we instead engaged a dedicated PPI lived-experience panel who we met with regularly throughout the study to seek their feedback on the process, specific pathway items and the pathway structure. We also had representatives from charities and support groups for TS as part of the expert consensus group and these individuals also attended the regional workshops.

## Conclusion

Through a Delphi survey of clinical and academic experts in tic disorders, regional workshops with HCPs and a PPI panel of parents of CYP with tics, we have designed an expert-recommended service pathway for the referral, assessment, treatment and monitoring of tics in CYP in England. The proposed service model provides clear, consensus-based guidance for clinicians on referral thresholds, evidence-based interventions and service configuration. Adoption of this model can enhance early identification, reduce misdiagnosis, support shared care planning and improve coordination between primary, neurodevelopmental and mental health services—ultimately leading to better outcomes for CYP with tic disorders and their families.

We have created a visual flow-chart showing the major points of the pathway and written accompanying guidance for HCPs, commissioners and service managers to aid implementation of the pathway. These documents are available through the study team who can provide support with implementation based on local context and service configuration. Future research is required to conduct an implementation-evaluation study to begin embedding the service pathway in existing services for CYP with mental health/neurodevelopmental difficulties including tics.

## Supplementary material

10.1136/bmjopen-2025-107534 online supplemental file 1

## Data Availability

All data relevant to the study are included in the article or uploaded as supplementary information.
