# Supplementary material for: Development of a tic service model for children and young people in England: a Delphi study
Source: BMJ Open. 2026 Apr 28;16(4):e107534. doi: 10.1136/bmjopen-2025-107534 (PMC13141126; doi:10.1136/bmjopen-2025-107534)
Supplement: online supplemental file 1 [file bmjopen-16-4-s001.pdf]

Supplemental Materials

For further information on the Delphi survey process and how the finalised items were transformed into service model features, please see Tables S1 and S2 below.

Table S1: Delphi Survey Items and Inclusions/Exclusions from each Survey Round

Table presenting all Delphi survey items, including ratings across all three rounds and outcomes (i.e., consensus reached for exclusion or inclusion within service model)

Item	Round 1	Round 2	Round 3	Outcome
Service Design/Structure				
1) The service should assess and diagnose tic disorders.	92% = Include, 2% = Exclude	96% = Include, 2% = Exclude		Approved in Round 2.
2) The service should provide treatment for tic disorders.	96% = Include, 0% = Exclude	98% = Include, 0% = Exclude		Approved in Round 2.
3) The service should monitor and review tic disorders.	58% = Include, 0% = Exclude	74% = Include, 0% = Exclude		Approved in Round 2.
4) The service should have an upper age limit with structured support for transition into adult services.	63% = Include, 12% = Exclude	69% = Include, 6% = Exclude	75% = Include, 4% = Exclude	Approved in Round 3.
5) Tic services should be embedded within a wider service model whereby additional co-morbidities can be assessed and treated.	78% = Include, 0% = Exclude	88% = Include, 0% = Exclude		Approved in Round 2.
6) A tic service should follow a hub and spoke model (i.e., where less specialist services (Tiers 1 & 2) refer into a regional specialist service (Tier 3).	New item added in Round 2	61% = Include, 10% = Exclude	69% = Include, 8% = Exclude	Item wording revised and approved at consensus meeting*. Revised item: "*There should be an option to refer complex cases to a specialist service where needed.*"
Referral				
7) The service should receive referrals from parents/carers.	28% = Include,	22% = Include,	16% = Include,	Item reviewed and rejected at consensus meeting.

	38% = Exclude	44% = Exclude	44% = Exclude	
8) The service should receive referrals from schools/education settings.	44% = Include, 38% = Exclude	42% = Include, 28% = Exclude	35% = Include, 28% = Exclude	Item reviewed and rejected at consensus meeting.
9) The service should receive referrals from GPs.	77% = Include, 2% = Exclude	82% = Include, 2% = Exclude		Approved in Round 2.
10) The service should receive referrals from secondary services (e.g., CAMHS, Paediatrics).	91% = Include, 0% = Exclude	97% = Include, 2% = Exclude		Approved in Round 2.
11) The service should receive referrals from third sector organisations (e.g., Tourettes Action).	42% = Include, 28% = Exclude	40% = Include, 26% = Exclude	34% = Include, 28% = Exclude	Item reviewed and rejected at consensus meeting.
12) Having tics for 6 months or more should be a referral criterion.	48% = Include, 30% = Exclude	51% = Include, 31% = Exclude	58% = Include, 33% = Exclude	Item wording revised and approved at consensus meeting. Additional revisions also made in consultation with clinical and PPI experts. Revised item: *"Having tics for 12 months or more and/or tics which have a significant impact should be a referral criterion."*
13) Having tics that cause impairment/distress to the child or young person should be a referral criterion.	65% = Include, 14% = Exclude	77% = Include, 12% = Exclude		Approved in Round 2.
Reviewing and Monitoring				
14) Within the agreed age range, the service should have no time limit for duration of care.	51% = Include, 12% = Exclude	60% = Include, 12% = Exclude	66% = Include, 6% = Exclude	Item reviewed and rejected at consensus meeting.
15) The decision to discharge from the service should be based on clinical	86% = Include, 4% = Exclude	91% = Include, 2% = Exclude		Approved in Round 2.

need, with provision to re-engage within a specified time frame.				
16) The service should write a brief report to the referring clinician to summarise findings and treatment options.	93% = Include, 2% = Exclude	98% = Include, 0% = Exclude		Approved in Round 2.
17) The service design should include clarity about follow-up care and who will provide this (e.g., shared care of medication prescriptions or monitoring with GPs, transfer to adult services, return of care to the referrer, etc.).	98% = Include, 0% = Exclude	100% = Include, 0% = Exclude		Approved in Round 2.
18) The service design should offer confirmation of tics and related needs for children and young people (CYP) to use to gain appropriate reasonable adjustments at school/education provisions.	New item added in Round 2	94% = Include, 4% = Exclude	98% = Include, 2% = Exclude	Approved in Round 3.
Professional Roles				
19) Paediatricians are essential when delivering a tic service.	54% = Include, 10% = Exclude	58% = Include, 8% = Exclude	64% = Include, 6% = Exclude	Item wording reviewed at consensus meeting and merged with items 20, 21 and 22. Revised item: *"A professional with appropriate medical training and expertise, e.g., Child and Adolescent Psychiatrist, Child and Adolescent Neuropsychiatrist and Paediatrician or similar."*
20) Paediatric Neurologists are essential when	59% = Include, 10% = Exclude	64% = Include, 12% = Exclude	69% = Include, 13% = Exclude	Item wording reviewed at consensus meeting and merged with items 19, 21 and 22. Revised item: *"A professional with*

delivering a tic service.				*appropriate medical training and expertise, e.g., Child and Adolescent Psychiatrist, Child and Adolescent Neuropsychiatrist and Paediatrician or similar."*
21) Child & Adolescent Psychiatrists are essential when delivering a tic service.	72% = Include, 8% = Exclude	75% = Include, 8% = Exclude		Item wording reviewed at consensus meeting and merged with items 19, 20 and 22. Revised item: *"A professional with appropriate medical training and expertise, e.g., Child and Adolescent Psychiatrist, Child and Adolescent Neuropsychiatrist and Paediatrician or similar."*
22) Child & Adolescent Neuropsychiatrists are essential when delivering a tic service.	54% = Include, 18% = Exclude	Edited to add clarification: Child & Adolescent Neuropsychiatrist (works with CYP with mental health conditions that have a neurological basis).<br><br>62% = Include, 12% = Exclude	71% = Include, 10% = Exclude	Item wording reviewed at consensus meeting and merged with items 19, 20 and 21. Revised item: *"A professional with appropriate medical training and expertise, e.g., Child and Adolescent Psychiatrist, Child and Adolescent Neuropsychiatrist and Paediatrician or similar."*
23) Clinical Psychologist/CBT Therapists are essential when delivering a tic service.	80% = Include, 4% = Exclude	89% = Include, 2% = Exclude		Item wording reviewed at consensus meeting and merged with item 27. Revised item: *"A professional with appropriate training and expertise in behavioural and psychological therapies, e.g., psychologist, nurse specialist or similar."*
24) Clinical Neuropsychologists are essential when delivering a tic service.	54% = Include, 18% = Exclude	29% = Include, 25% = Exclude	25% = Include, 21% = Exclude	Item reviewed and rejected at consensus meeting.

25) Counselling Psychologists are essential when delivering a tic service.	22% = Include, 34% = Exclude	23% = Include, 38% = Exclude	18% = Include, 27% = Exclude	Item reviewed and rejected at consensus meeting.
26) Educational Psychologists are essential when delivering a tic service.	20% = Include, 36% = Exclude	18% = Include, 39% = Exclude	12% = Include, 45% = Exclude	Item reviewed and rejected at consensus meeting.
27) Nurse Specialists are essential when delivering a tic service.	55% = Include, 12% = Exclude	63% = Include, 12% = Exclude	64% = Include, 8% = Exclude	Item wording reviewed at consensus meeting and merged with item 23. Revised item: *"A professional with appropriate training and expertise in behavioural and psychological therapies, e.g., psychologist, nurse specialist or similar."*
28) Speech and Language Therapists are essential when delivering a tic service.	10% = Include, 44% = Exclude	10% = Include, 50% = Exclude	4% = Include, 59% = Exclude	Item reviewed and rejected at consensus meeting.
29) Occupational Therapists are essential when delivering a tic service.	30% = Include, 34% = Exclude	17% = Include, 37% = Exclude	10% = Include, 39% = Exclude	Item reviewed and rejected at consensus meeting.
30) Physiotherapists are essential when delivering a tic service.	14% = Include, 46% = Exclude	8% = Include, 48% = Exclude	4% = Include, 45% = Exclude	Item reviewed and rejected at consensus meeting.
31) Other (please state and then rate).			Removed after Round 1	Removed after Round 1.
A Professional with high level of knowledge and experience regarding common comorbidities and treatment. (item suggested by participant in Round 1)	100% = Include, 0% = Exclude (rated by participant who added item)	Decision made by study team not to include		Rejected after Round 1.
Training, Supervision & Development				

32) Designated clinicians working in local services for children and young people should have sufficient training to assess/identify tic disorders.	96% = Include, 0% = Exclude	100% = Include, 0% = Exclude		Approved in Round 2.
33) Designated clinicians working in local services for children and young people should have sufficient training to diagnose tic disorders.	79% = Include, 8% = Exclude	89% = Include, 4% = Exclude		Approved in Round 2.
34) Designated clinicians working in local services for children and young people should have sufficient training to monitor tic disorders.	75% = Include, 0% = Exclude	89% = Include, 0% = Exclude		Approved in Round 2.
35) Each local service for CYP should have a minimum number of clinicians who have received sufficient training to treat tic disorders.	81% = Include, 6% = Exclude	92% = Include, 4% = Exclude		Approved in Round 2.
36) Protected time for specialist supervision should be available for practitioners providing treatment for tics.	65% = Include, 2% = Exclude	77% = Include, 0% = Exclude		Approved in Round 2.
Primary Care				
37) Primary care practitioners (i.e., GPs) should have sufficient training to assess/identify tic disorders in CYP.	75% = Include, 6% = Exclude	87% = Include, 2% = Exclude		Approved in Round 2.
38) Primary care practitioners should have sufficient	85% = Include,	96% = Include,		Approved in Round 2.

training to make appropriate referrals for tic disorders in CYP.	0% = Exclude	0% = Exclude		
39) Primary care practitioners should have sufficient training to diagnose simple tic disorders in CYP.	40% = Include, 20% = Exclude	42% = Include, 15% = Exclude	34% = Include, 10% = Exclude	Item reviewed and rejected at consensus meeting.
40) Primary care practitioners should have sufficient training on managing shared care protocols for tic disorders in CYP.	79% = Include, 2% = Exclude	92% = Include, 0% = Exclude		Approved in Round 2.
General				
41) Professional accrediting organisations (e.g., the Royal Colleges, British Psychological Society, the Health and Care Professions Council) should include training in mandated topics on tic disorders in CYP.	72% = Include, 6% = Exclude	77% = Include, 4% = Exclude		Approved in Round 2.
42) Clinicians working in a tic service should be provided opportunities for continuous professional development (CPD) to develop their knowledge and skills in clinical research.	79% = Include, 0% = Exclude	83% = Include, 0% = Exclude		Item wording revised and approved at consensus meeting. Revised item: *"Clinicians working in the service should be allocated time for continued professional development (CPD) and to engage in activities related to audit, service evaluation and clinical research."*
43) Clinicians working in a tic service should be allocated protected time to support with the delivery of clinical research.	46% = Include, 4% = Exclude	40% = Include, 4% = Exclude	31% = Include, 4% = Exclude	Item wording revised and approved at consensus meeting. Revised item: *"The service should be audited to identify areas of improvement, in alignment with the NICE Quality Standards Framework.* *Further edited by study team:*

				The service should be audited to identify areas of improvement, to align with the NICE Quality Standards Framework."
44) Quality Network for Care Quality Commission (CQC) inspections and ratings of local health services should include consideration of tic services for CYP.	78% = Include, 4% = Exclude	83% = Include, 2% = Exclude		Approved in Round 2.
45) Services should add tic disorders to their standard operating procedures.	86% = Include, 0% = Exclude	94% = Include, 0% = Exclude		Approved in Round 2.
Funding				
46) All Integrated Care Boards and/or Health and Care Partnerships should commission and appropriately fund services to assess, treat, diagnose and monitor tic disorders in CYP.	93% = Include, 0% = Exclude	96% = Include, 0% = Exclude		Approved in Round 2.
47) The tic service should be a nationally commissioned service.	47% = Include, 20% = Exclude	58% = Include, 18% = Exclude	64% = Include, 21% = Exclude	Item reviewed and rejected at consensus meeting.
48) Tic services should be commissioned and organised locally by each ICB.	59% = Include, 6% = Exclude	68% = Include, 4% = Exclude	83% = Include, 2% = Exclude	Approved in Round 3.
Assessment (what tools?)				
49) The service should use a standardised taxonomy (e.g., DSM-5 or ICD-11) to assess tic disorders in CYP.	80% = Include, 4% = Exclude	91% = Include, 0% = Exclude		Approved in Round 2.
50) The service should use general	84% = Include,	91% = Include,		Approved in Round 2.

clinical assessment (e.g., interview) to assess tic disorders in CYP.	2% = Exclude	0% = Exclude		
51) The service should include physical and neurological examination when assessing tic disorders in CYP.	70% = Include, 4% = Exclude	90% = Include, 2% = Exclude		Approved in Round 2.
52) The service should use standardised measures (e.g., the Yale Global Tic Severity Scale, Premonitory Urges for Tics Scale, Parent Tic Questionnaire) to assess CYP with tics.	76% = Include, 4% = Exclude	83% = Include, 4% = Exclude		Approved in Round 2.
Treatment Options				
53) The service should offer psychoeducation for CYP, families and schools.	97% = Include, 0% = Exclude	100% = Include, 0% = Exclude		Approved in Round 2.
54) The service should offer HRT (Habit Reversal Therapy)/CBIT (Comprehensive Behavioural Intervention for Tics) as a behavioural therapy.	83% = Include, 2% = Exclude	88% = Include, 0% = Exclude		Item wording reviewed at consensus meeting and merged with items 55 and 57. Revised item: *"The service should offer appropriate evidence-based psychological therapies (e.g., HRT, CBIT, ERP)."*
55) The service should offer ERP (Exposure and Response Prevention) as a behavioural therapy.	74% = Include, 2% = Exclude	83% = Include, 0% = Exclude		Item wording reviewed at consensus meeting and merged with items 54 and 57. Revised item: *"The service should offer appropriate evidence-based psychological therapies (e.g., HRT, CBIT, ERP)."*
56) The service should offer an online	59% = Include,	65% = Include,	71% = Include,	Approved in Round 3.

delivery option as a behavioural therapy.	8% = Exclude	4% = Exclude	4% = Exclude	
57) The service should offer Psychological Therapies, such as CBT (cognitive behavioural therapy) and third wave interventions.	64% = Include, 6% = Exclude	81% = Include, 4% = Exclude		Item wording reviewed at consensus meeting and merged with items 54 and 55. Revised item: *"The service should offer appropriate evidence-based psychological therapies (e.g., HRT, CBIT, ERP)."*
58) The service should offer pharmacotherapy (medication).	79% = Include, 4% = Exclude	94% = Include, 4% = Exclude		Approved in Round 2.
59) The service should offer specialist training and consultation clinics for community clinicians and schools (e.g., staff and teachers).	76% = Include, 4% = Exclude	79% = Include, 4% = Exclude		Approved in Round 2.
60) The service should offer treatment should be available remotely via teleconference.	44% = Include, 10% = Exclude	48% = Include, 10% = Exclude	58% = Include, 6% = Exclude	Item wording revised and approved at consensus meeting. Revised item: *"Treatment should be available remotely via teleconference where clinically appropriate."*
61) The service should offer pain management.	47% = Include, 22% = Exclude	54% = Include, 21% = Exclude	56% = Include, 17% = Exclude	Item wording reviewed at consensus meeting. Merged with item 62 and included in broad clinical role descriptions. Revised item: *"The service should provide access to a professional with appropriate training and expertise in pain management and/or physiotherapy."*
62) The service should offer physiotherapy.	20% = Include, 34% = Exclude	14% = Include, 39% = Exclude	10% = Include, 41% = Exclude	Item wording reviewed at consensus meeting. Merged with item 61 and included in broad clinical role descriptions. Revised item: *"The service should provide access to a professional with appropriate training and expertise in pain management and/or physiotherapy."*

63) The service should offer group treatments, in addition to individually delivered treatment, should be available.	40% = Include, 20% = Exclude	45% = Include, 21% = Exclude	41% = Include, 15% = Exclude	Item wording revised and approved at consensus meeting. Revised item: *"Group treatment should be available where appropriate."*

**No items were revised between rounds. Any revisions to the item wording occurred during the consensus meeting and recorded accordingly*

Table S2: Transformation of Delphi Survey Consensus Items into Service Model Pathway Features and Structure

Delphi Consensus Items*	Service Model (Pathway) Specification
**Service Design/Structure**	
1. The service should assess and diagnose tic disorders.<br>2. The service should provide treatment for tic disorders.<br>3. The service should monitor and review tic disorders.<br>4. The service should have an upper age limit with structured support for transition into adult services.<br>5. Tic services should be embedded within a wider service model whereby additional co-morbidities can be assessed and treated.	The tic disorder pathway should be offered within neurodevelopmental or children and young people's mental health services (e.g. Paediatrics or CAMHS, depending on local service configuration). This is to ensure that provision for tic disorders is embedded within a wider service model whereby additional co-morbidities can be assessed and treated.<br><br>Professionals working within these services should also have the relevant expertise in child development and/or mental health.
**Referral**	
6. There should be an option to refer complex cases to a specialist service where needed.<br>9. The service should receive referrals from GPs.<br>10. The service should receive referrals from secondary services (e.g., CAMHS, Paediatrics).	Referrals can be made by a GP OR via appropriate secondary/specialist services.<br><br>Electronic referral via standardised referral form.<br><br>Referral from GPs is recommended to ensure red flags can be checked.

	In rare cases, tics may be a sign of another neurological abnormality and require immediate and urgent referral to neurology. Please see NICE guideline NG127: Recommendations for children aged under 16 | Suspected neurological conditions: recognition and referral | Guidance | NICE.<br><br>Referral from secondary services is recommended in cases where tics are identified during screening or assessment of an ND or MH condition.
12. Having tics for 6 months or more should be a referral criterion. → Having tics for 12 months or more and/or tics which have a significant impact should be a referral criterion. (Following input from PPI experts, referral criterion for duration of tics revised)<br>13. Having tics that cause impairment/distress to the child or young person should be a referral criterion.	If any of the below questions are true, then refer:<br><br>Have the tics been present for 12 months or more?<br><br>Are the tics causing psychological or physical distress or harm?<br><br>Are the tics causing functional impairment?<br><br>**If 'Yes' to any of these questions, refer immediately**<br><br>If child does not meet any of these criteria, signpost to Tourettes Action.<br><br>Advise to watch/wait and come back if symptoms persist or worsen.
**Assessment**	
5. Tic services should be embedded within a wider service model whereby additional co-morbidities can be assessed and treated.	Liaison with other services:<br><br>If concerns that tics are caused by another neurological condition, refer to/liaise with appropriate service.<br><br>If ADHD/ASD assessment required, refer to/liaise with NDD service.<br><br>If mental health difficulties are severe, refer to/liaise with CAMHS.<br><br>If tics likely to be functional, refer to/liaise with CAMHS.<br><br>If pain needs management refer to/liaise with specialist.<br><br>If medication required for tics, ADHD, or mental health, refer to/liaise with a medically trained professional (psychiatrist, paediatrician, nurse prescriber).

	Liaison can take the form of a regular multidisciplinary team meeting OR a clear, well-specified arrangement for communication and consultation between professionals working in each service.
49. The service should use a standardised taxonomy (e.g., DSM-5 or ICD-11) to assess tic disorders in CYP.	Diagnostic decision in accordance with DSM-5 or ICD-11 criteria.
50. The service should use general clinical assessment (e.g., interview) to assess tic disorders in CYP.	Interview with child/young person and parent/carer.
51. The service should include physical and neurological examination when assessing tic disorders in CYP.	Physical & neurological examination.
52. The service should use standardised measures (e.g., the Yale Global Tic Severity Scale, Premonitory Urges for Tics Scale, Parent Tic Questionnaire) to assess CYP with tics.	Yale Global Tic Severity Scale.<br><br>Parent Tic Questionnaire.
**Treatment, Management and Support**	
53. The service should offer psychoeducation for CYP, families and schools.<br>59. The service should offer specialist training and consultation clinics for community clinicians and schools (e.g., staff and teachers).	Psychoeducation available (all cases) for young person and family and other relevant parties (e.g., school).
54. The service should offer HRT (Habit Reversal Therapy)/CBIT (Comprehensive Behavioural Intervention for Tics) as a behavioural therapy.<br>55. The service should offer ERP (Exposure and Response Prevention) as a behavioural therapy.<br>57. The service should offer Psychological Therapies, such as CBT (cognitive behavioural	Evidence-based psychological therapies (e.g., ERP, HRT, CBIT) available in online, group or one-to-one format, in-person or remotely via teleconference if clinically appropriate.

therapy) and third wave interventions.<br>• Items 54, 55 & 57 merged to create final item → The service should offer appropriate evidence-based psychological therapies (e.g., HRT, CBIT, ERP).<br>56. The service should offer an online delivery option as a behavioural therapy.<br>60. Treatment should be available remotely via teleconference where clinically appropriate.<br>63. Group treatment should be available where appropriate.	
58. The service should offer pharmacotherapy (medication).	Pharmacotherapy available alone or in conjunction with psychological therapy.
61. The service should offer pain management.<br>62. The service should offer physiotherapy.<br>• Items 61 & 62 merged to create final item → The service should provide access to a professional with appropriate training and expertise in pain management and/or physiotherapy.	If pain needs management refer to specialist clinic or professional with relevant expertise.
**Reviewing, Monitoring, and Follow-up**	
15. The decision to discharge from the service should be based on clinical need, with provision to re-engage within a specified time frame.	Discharge from tic disorder pathway when the tics are well-managed (based on clinical symptoms and discussion with CYP and family).<br><br>Patient Initiated Follow-Up (PIFU) within specified time-frame (discharge letter should detail how to re-engage and the PIFU time-frame).
16. The service should write a brief report to the referring clinician to summarise findings and treatment options.<br>18. The service design should offer confirmation of tics and related needs for children and young people (CYP) to use to gain	Report summarising diagnosis and treatment plan shared with referring clinician, young person & appropriate agencies in accordance with service protocols & Data Protection regulations.<br><br>The report should be shared with appropriate agencies including the Special Educational Needs Coordinator at the child's school, if consent has been given by the parent/carer

appropriate reasonable adjustments at school/education provisions.	(in accordance with the service's existing protocols for data sharing).
17. The service design should include clarity about follow-up care and who will provide this (e.g., shared care of medication prescriptions or monitoring with GPs, transfer to adult services, return of care to the referrer, etc.)	Shared care arrangements, if applicable, must be specified in the discharge letter.<br><br>If shared care agencies are unwilling to accept the transfer of care, the child/young person must remain within the tic service.
**Workforce Planning, Training and Professional Development*** **	
19. Paediatricians are essential when delivering a tic service.<br>20. Paediatric Neurologists are essential when delivering a tic service.<br>21. Child & Adolescent Psychiatrists are essential when delivering a tic service.<br>22. Child & Adolescent Neuropsychiatrists are essential when delivering a tic service.<br>• Items 19, 20, 21 & 22 synthesised and summarised to create a new item → The tic service must include a professional with appropriate medical training and expertise, e.g., Child and Adolescent Psychiatrist, Child and Adolescent Neuropsychiatrist and Paediatrician or similar.<br>32. Designated clinicians working in local services for children and young people should have sufficient training to assess/identify tic disorders.<br>33. Designated clinicians working in local services for children and young people should have sufficient training to diagnose tic disorders.<br>34. Designated clinicians working in local services for children and young people should have sufficient training to monitor tic disorders.<br>35. Each local service for CYP should have a minimum number of clinicians who have received sufficient training to treat tic disorders.	
23. Clinical Psychologist/CBT Therapists are essential when delivering a tic service.<br>27. Nurse Specialists are essential when delivering a tic service.<br>• Items 23 & 27 synthesised and summarised to create a new item → A professional with appropriate training and expertise in behavioural and psychological therapies, e.g., psychologist, nurse specialist or similar.<br>35. Each local service for CYP should have a minimum number of clinicians who have received sufficient training to treat tic disorders.	
37. Primary care practitioners (i.e., GPs) should have sufficient training to assess/identify tic disorders in CYP.<br>38. Primary care practitioners should have sufficient training to make appropriate referrals for tic disorders in CYP.<br>40. Primary care practitioners should have sufficient training on managing shared care protocols for tic disorders in CYP.	
36. Protected time for specialist supervision should be available for practitioners providing treatment for tics.	

41. Professional accrediting organisations (e.g., the Royal Colleges, British Psychological Society, the Health and Care Professions Council) should include training in mandated topics on tic disorders in CYP.
**Funding*** **
46. All Integrated Care Boards and/or Health and Care Partnerships should commission and appropriately fund services to assess, treat, diagnose and monitor tic disorders in CYP.<br>48. Tic services should be commissioned and organised locally by each ICB.
**General*** **
42. Clinicians working in the service should be allocated time for continuous professional development (CPD) and to engage in activities related to audit, service evaluation and clinical research.<br>43. The service should be audited to identify areas of improvement, to align with the NICE Quality Standards Framework.<br>44. Quality Network for Care Quality Commission (CQC) inspections and ratings of local health services should include consideration of tic services for CYP.<br>45. Services should add tic disorders to their standard operating procedures.

CAMHS = Child and Adolescent Mental Health Services; CBIT= Comprehensive Behavioural Intervention for Tics; CBT = Cognitive Behavioural Therapy; CYP = Children & Young People; DSM-5 = Diagnostic and Statistical Manual of Mental Disorders, Fifth Edition; ERP = Exposure Response Prevention; GP = General Practitioner; HRT = Habit Reversal Therapy; ICD-11 = International Classification of Diseases, Eleventh Revision; MH = Mental Health; NICE = National Institute of Health & Care Excellence; ND = Neurodevelopmental; NDD = Neurodevelopmental Disorder; PPI = Patient and Public Involvement; PTQ = Parent Tic Questionnaire; YGTSS = Yale Global Tic Severity Scale

*The ordering of Delphi Consensus items does not always follow the ordering of items in the original survey to better show how the items align with the features of the pathway. The original Delphi item numbers have been included next to the items and notes added where these have been revised or merged with other items.

**Items 5 and 35 have been repeated because they informed more than one area of the pathway.

***Items relating to broader context (Workforce Planning, Training and Professional Development, Funding and General) do not link to one specific part of the pathway and are designed to inform the overall context and fit of the pathway within local service structures.
