# Supplementary material for: Development of a tic service model for children and young people in England: a Delphi study
Source: BMJ Open. 2026 Apr 28;16(4):e107534. doi: 10.1136/bmjopen-2025-107534 (PMC13141126; doi:10.1136/bmjopen-2025-107534)
Supplement: Supplementary file 2 [file bmjopen-2025-107534.reviewer_comments.pdf]

PEER REVIEW HISTORY

BMJ Open publishes all reviews undertaken for accepted manuscripts. Reviewers are asked to complete a checklist review form and are provided with free text boxes to elaborate on their assessment. These free text comments are reproduced below.

ARTICLE DETAILS

Title (Provisional)

Development of a Tic Service Model for Children and Young People in England: A Delphi study

Authors

Hall, Sophie Susannah; Rattu, Nikita R; Hall, Charlotte Lucy; KILGARIFF, JOSEPH; Murphy, Tara; James, Nadya; McNally, Emma; Rimmer, Suzanne; Thomson, Louise; Groom, Maddie

---

VERSION 1 - REVIEW

**Reviewer**	**1**
**Name**	**Ludlow, Amanda**
**Affiliation**	**University of Hertfordshire**
**Date**	**26-Sep-2025**
**COI**	**None**

The article is the first to offer a consensus view of health professionals in establishing guidelines for tic pathway in children.

The introduction is clearly written, includes relevant literature and guides the reader as to the vital need for clear pathway for offering diagnostic assessment, treatment and management of tics in England.

The Delphi rounds are nicely presented, and tables help to inform how the items were developed over the 3 rounds of the survey.

Some minor comments:

Methods:

Could provide a couple of sentences in main text on their ethics particularly around the welfare of experts. Be explicit whether NHS trusts were recruited from and why university ethics was appropriate here.

Discussion:

Need to acknowledge in the discussion the limitation of recruiting by email invitation only, particularly with the inclusion of many of the contacts known to the study team already.

It would be good to include some context around how the pathway might work in practice depending on whether the service fits within existing neurodevelopmental/mental health services. Whilst I appreciate that this would be driven by local context and service design within specific ICBs and Places, are there any examples of how this would work using these services and how might this differ on the advice being given.

**Reviewer**	**2**
**Name**	**Young, Bryan**
**Affiliation**	**Douglas Mental Health University Institute**
**Date**	**24-Dec-2025**
**COI**	**None**

Thank you for the opportunity to review this manuscript. I enjoyed reading about your approach to creating a care pathway for children and young people in the United Kingdom who present with tic disorders, including Tourette's syndrome. It was a well-written paper, and I especially appreciated your efforts to develop a pathway via consensus measures.

Below are my comments and recommendations in no particular order for your consideration:

Comment #1:
I believe the manuscript could be improved by enhancing clarity regarding the concurrent efforts to reach consensus. I found myself rereading the Stage 1 and Stage 2 sections of the Methods to better understand the timeline and interconnections of these initiatives. As I understand it, while the Delphi process was ongoing, regional workshops were also held, and these workshops, in addition to identifying implementation barriers, also served to refine the final wording of the care pathway standard.

My recommendation is to revise the Delphi Survey Process figure to better illustrate the role of the regional workshops. It would be helpful to present this information in a linear format, with dates, so readers can more clearly see how the two activities relate to each other.

Comment #2:
I appreciated your commitment to engaging patients and parents throughout your work. However, providing more clarity on how you define lived experience, and your decision to exclude children and young people with lived experience of tic disorders from the Delphi consensus process would enhance the manuscript. Early in the paper, you state that patients and parents/caregivers were involved in the design, conduct, reporting, and dissemination plans of your research. Later in your Discussion, you mention that children and young people with lived experience of tic disorders were excluded from the Delphi due to the terminology and

language used in the care pathway standard. It would be helpful to align these statements and provide further explanation of the decision-making process for forming your Patient and Public Involvement (PPI) panel, as well as the role of lived experience expertise in the Delphi process itself.

Comment #3:
As is often the case with Delphi manuscripts, displaying the variations in specific clauses and their wording between rounds can be challenging. While I appreciated the efforts to showcase some of these alterations in Table S1: Delphi Survey Items and Inclusions/Exclusions from Each Survey Round, I believe the manuscript would benefit from a clearer table that outlines how each item evolved from one round to the next.

For instance, displaying wording for each item per round, to showcase how it evolved over time (from the original wording to its final form). Additionally, showing all the items grouped together and reworded would enhance readability, as I found it difficult to track which clause items were combined. Additionally, I would like to gain a better understanding of the discussions that took place during the Consensus Meeting. Was there a specific set of criteria used to determine which items could be consolidated? What feedback was received, and how did this ultimately lead to the condensing of clauses?

Comment #4:
Table 1 presents the demographics of the 49 Delphi participants. To enhance readers' understanding of voting patterns across stakeholder groups, it would be helpful to clarify how votes were split throughout the Delphi process. For instance, did researchers demonstrate a strong preference for a specific item while healthcare professionals (HCPs) did not find it valuable? If this information was collected, I recommend including a supplemental table to illustrate the voting distribution by stakeholder group. This could then be further expanded upon in your Discussion section.

For example, you mentioned efforts to introduce additional referral criteria beyond the 12-month benchmark for symptoms, including their functional impact and the psychological or physical harm/distress. Did this discussion originate in the Delphi process? If so, what was the voting distribution by demographic? In other words, who valued this inclusion, and how did you ultimately decide to incorporate it into the final version of the pathway with your PPI panel?

Additional Comment: Re: Table 1
From my view of the paper, part of the table appears to be cut off. Ensure all genders are represented on the table, as currently, I only see Female 69.4% (34), 68.8% (33).

Final Comments:
I truly appreciate the work done in this manuscript to better outline and introduce a specific pathway for children and young people with tic disorders or symptoms. This effort is greatly needed, and I value your time and work you have put into this research, and subsequent work

to support implementation and evaluation. Thank you for the opportunity to review this manuscript.

VERSION 1 - AUTHOR RESPONSE

Reviewer: 1: Dr. Amanda Ludlow, University of Hertfordshire

1. Methods: Could provide a couple of sentences in main text on their ethics particularly around the welfare of experts. Be explicit whether NHS trusts were recruited from and why university ethics was appropriate here.

➢ Thank you for highlighting this point. We have added further detail on ethical procedures on page 19:

"The study involved only adult experts participating in their professional roles, with no involvement of NHS patients or access to patient data. Therefore, NHS REC review was not required under HRA guidance, and approval was appropriately granted by the University of Nottingham Faculty of Medicine and Health Sciences Research Ethics Committee (FMHS 313-0623). Although some participants were employed by NHS organisations, no NHS Trusts were recruited as research sites. Participant welfare was protected through informed consent, anonymisation of Delphi responses, and non-identifiable note-taking during workshops, with the option to withdraw at any time."

2. Discussion: Need to acknowledge in the discussion the limitation of recruiting by email invitation only, particularly with the inclusion of many of the contacts known to the study team already.

➢ Thank you for the opportunity to clarify this point. We created the study contacts through internet searching/discussion with our wider study group. Therefore, not all contacts were not directly known by the team. We have revised the manuscript accordingly on page 6-7:

"*Participants were invited via email, starting with a list of experts identified by the study team through publicly available information (e.g., clinicians known to have specialist expertise in tics and academics with peer reviewed publications in this field). Additional participants were then recruited through snowball sampling from this initial group."*

We have also reflected on this in the discussion on page 16:

"Recruitment for the Delphi was conducted via email, beginning with experts identified through publicly available information; although many invitees were not personally known to the team and snowball sampling widened reach, this approach may still have excluded individuals less visible within established networks."

3. It would be good to include some context around how the pathway might work in practice depending on whether the service fits within existing neurodevelopmental/mental health services. Whilst I appreciate that this would be driven by local context and service design within specific ICBs and Places, are there any examples of how this would work using these services and how might this differ on the advice being given.

➢ In practice, the pathway is intended to be embedded within existing local service structures. In some areas, tic disorders are managed within neurodevelopmental teams, where assessments for co-occurring conditions such as ADHD or autism already sit, and multidisciplinary input is routine. In other areas, tic management is

located within children and young people's mental health services, where behavioural interventions may be delivered by psychological therapy teams, with paediatric or neurology input accessed via shared-care arrangements. These differences affect which team leads specific components of the pathway rather than the content of the pathway itself, and the model was designed to accommodate this variation across healthcare boards. We are currently conducting further work to prepare for implementation in a number of services across England and identifying how best to implement the pathway according to different service structures. We have included reference to this in the manuscript – see P15.

"We are currently developing plans for further research to implement and evaluate the pathway in services in England, including how best to implement the pathway in different service structures."

Reviewer: 2: Mr. Bryan Young, Douglas Mental Health University Institute

1. I believe the manuscript could be improved by enhancing clarity regarding the concurrent efforts to reach consensus. I found myself rereading the Stage 1 and Stage 2 sections of the Methods to better understand the timeline and interconnections of these initiatives. As I understand it, while the Delphi process was ongoing, regional workshops were also held, and these workshops, in addition to identifying implementation barriers, also served to refine the final wording of the care pathway standard. My recommendation is to revise the Delphi Survey Process figure to better illustrate the role of the regional workshops. It would be helpful to present this information in a linear format, with dates, so readers can more clearly see how the two activities relate to each other.

- We have revised the description of the workshops to make it clearer that the workshop participants did not directly review or edit the wording of the pathway items; rather, the workshop discussion points provided the research team with more in-depth knowledge of healthcare services for CYP. This knowledge was reflected in how we developed the consensus items into the care pathway. We have included this text on page 9.

  "The knowledge gained during the workshops helped us to word the pathway features (the final wording of the items approved through the Delphi) to enhance future acceptability/implementation in practice. The Delphi items themselves were not presented or discussed at the workshops."
  We have also made revisions to figure 1 as suggested.

2. I appreciated your commitment to engaging patients and parents throughout your work. However, providing more clarity on how you define lived experience, and your decision to exclude children and young people with lived experience of tic disorders from the Delphi consensus process would enhance the manuscript. Early in the paper, you state that patients and parents/caregivers were involved in the design, conduct, reporting, and dissemination plans of your research. Later in your Discussion, you mention that children and young people with lived experience of tic disorders were excluded from the Delphi due to the terminology and language used in the care pathway standard. It would be helpful to align these statements and provide further explanation of the decision-making process for forming your Patient and Public Involvement (PPI) panel, as well as the role of lived experience expertise in the Delphi process itself.

- We have clarified this in the manuscript under the PPI heading:

“Patient and public Involvement was embedded throughout the study. Lived experience was defined as being a parent or carer of a child or young person with a tic disorder and having experience of accessing healthcare for their child/young person’s tics. A PPI panel (n=10) was formed after advertising nationally. The panel comprised mothers (n=8) and fathers, was mostly white British (n=8) with 2 members from ethnic minority groups, recruited from 10 different regions of England. The panel was chaired by a parent of a young person with TS who also has a professional role as the CEO of the national charity, Tourettes Action (co-author EM). The PPI panel met every few months to review the study design (including some aspects published previously), share their own experience of healthcare pathways for CYP with tics and, in the later stages of the study, to refine the overall pathway structure and design. They also co-created dissemination materials to share the findings with the lived experience community.-design terminology used across the 61 items were not appropriate for younger participants. Instead, the PPI panel contributed meaningfully to the design, refinement, and interpretation of the pathway by reviewing the study aims, advising on clarity of language, and providing iterative feedback on the full set of consensus-derived items following the workshop. Their input shaped key components of the pathway

We did not recruit parents/carers or CYP with tic disorders to participate in the Delphi consensus process because the technical language and service design terminology used across the 61 items required a high-level understanding of healthcare pathways. Instead, the PPI panel described above and lived experience representatives from charities and support groups for Tourette Syndrome contributed to the refinement and overall structure of the pathway following the consensus workshop. Their input shaped key components of the pathway, including the referral criteria and the centrality of multi-disciplinary liaison between services. Charity and support group representatives (including the PPI panel chair) attended the regional workshops .”

- We have also reflected on this in the discussion, page 16:

“Finally, although lived experience expertise was central to the study, neither CYP with tic disorders nor their parents were included directly in the Delphi consensus survey. The technical and service configuration terminology of the Delphi items was not accessible to younger participants or those without clinical-academic expertise, and inclusion risked misinterpretation, and/or having to word the items in a way that limited their relevance to healthcare professionals and service pathway design. To ensure lived experience informed the process in a meaningful way, we instead engaged a dedicated PPI lived-experience panel who we met with regularly throughout the study to seek their feedback on the process, specific pathway items and the pathway structure. We also had representatives from charities and support groups for Tourette Syndrome as part of the expert consensus group and these individuals also attended the regional workshops.”

3. As is often the case with Delphi manuscripts, displaying the variations in specific clauses and their wording between rounds can be challenging. While I appreciated the efforts to showcase some of these alterations in Table S1: Delphi Survey Items and Inclusions/Exclusions from Each Survey Round, I believe the manuscript would benefit from a clearer table that outlines how each item evolved from one round to the next. For instance, displaying wording for each item per round, to showcase how it evolved over time (from the original wording to its final form). Additionally, showing all the items grouped together and reworded would enhance readability, as I found it difficult to track which clause items were combined.

- Thank you for highlighting this important point. The Delphi items didn’t change between rounds. There were two items added between rounds 1 and 2 (these are described as new items under the ‘Round 1’ column in Table S1) but no other item or

wording changes were made between rounds. We have made this clearer in our manuscript on page 10 as follows:

"*During the consensus meeting, these issues were considered in detail, and 10 items were revised—either merged with existing agreed items or re-worded to improve clarity—resulting in three new consolidated items. No items were revised between survey rounds."*

A footnote has been added to Table S1:

"No items were revised between rounds. Any revisions to the item wording occurred during the consensus meeting and recorded accordingly."

- In addition, the merging and rewording of specific items (in the consensus workshop process) are shown in Table S2. For example, under 'Workforce Planning, training and Professional Development', there is a specific point about items 19-22: •

"Items 19, 20, 21 & 22 synthesised and summarised to create a new item → The tic service must include a professional with appropriate medical training and expertise, e.g., Child and Adolescent Psychiatrist, Child and Adolescent Neuropsychiatrist and Paediatrician or similar."

We hope this makes it clear how some of the Delphi consensus items were merged or adjusted during the consensus meeting.

Additionally, I would like to gain a better understanding of the discussions that took place during the Consensus Meeting. Was there a specific set of criteria used to determine which items could be consolidated? What feedback was received, and how did this ultimately lead to the condensing of clauses?

- Consensus meetings were not audio-recorded or transcribed, which is important to note when responding to this comment, we have clarified this in the manuscript – Page 8:

"Discussions in the consensus workshop were documented using structured, outcome-focused notes rather than audio recordings, reflecting standard practice in many consensus workshops-focused notes rather than audio recordings, reflecting standard practice in many consensus workshops"

We have reflected on this as a limitation in the discussion:

"*Another limitation is that discussions during the consensus workshop were captured through structured, outcome-focused notetaking rather than audio recording. While this approach is widely used in consensus-building exercises, it may have resulted in the loss of some nuance in participants' discussions-focused note-taking rather than audio recording. While this approach is widely used in consensus-building exercises, it may have resulted in the loss of some nuance in participants' discussions"*

Nonetheless, we have provided clarity on the discussions within the manuscript as follows:

"*The 22 items that had not reached consensus by the end of Round 3 were discussed in a consensus meeting, where attendees reviewed each item and then voted to 'include' or 'exclude' it from the service model. As part of this discussion, the attendees also considered free-text feedback provided by the Delphi participants in each round of the survey. This feedback indicated that several of these non-consensus items contained overlapping*

content, ambiguous wording, or referenced specific professional roles rather than focusing on the underlying responsibilities of professionals delivering a tic service. During the consensus meeting, these issues were considered in detail, and 10 items were revised—either merged with existing agreed items or re-worded to improve clarity—resulting in three new consolidated items. Using the same ≥70% agreement criteria applied in the Delphi survey, all three revised items reached consensus for inclusion and another 12 items from the initial set of 22 non-consensus items were excluded, yielding a final set of 43 items."

Following the consensus meeting, the final items underwent further iterative review with the study team and PPI panel. This process resulted in additional refinements to improve clarity and applicability across different service structures. Similarly, feedback from the PPI panel highlighted the need for greater clarity in the referral criteria. While confirming a 12-month duration of tics is often required for diagnosis, families stressed that significant distress, harm, or functional impairment should also trigger referral. In response, the referral criteria were revised to specify that any one of these indicators (12-month duration, distress/harm, or impairment) should prompt referral."

4. Table 1 presents the demographics of the 49 Delphi participants. To enhance readers' understanding of voting patterns across stakeholder groups, it would be helpful to clarify how votes were split throughout the Delphi process. For instance, did researchers demonstrate a strong preference for a specific item while healthcare professionals (HCPs) did not find it valuable? If this information was collected, I recommend including a supplemental table to illustrate the voting distribution by stakeholder group. This could then be further expanded upon in your Discussion section. For example, you mentioned efforts to introduce additional referral criteria beyond the 12-month benchmark for symptoms, including their functional impact and the psychological or physical harm/distress. Did this discussion originate in the Delphi process? If so, what was the voting distribution by demographic? In other words, who valued this inclusion, and how did you ultimately decide to incorporate it into the final version of the pathway with your PPI panel?

➢ Thank you for this thoughtful suggestion. We agree that exploring voting patterns across stakeholder groups can be informative in some Delphi studies. However, in our study this analysis is not possible, and we have now clarified this in the manuscript. First, all Delphi responses were collected fully anonymously. Although demographic information (e.g., stakeholder group, region, professional role) was collected in aggregate form at baseline, these data were not linked to individual item-level ratings, in line with standard Delphi methodology that emphasises anonymity to minimise social pressure, reduce influence of dominant voices, and protect participants when expressing minority or critical views. As a result, it is not possible to reconstruct voting patterns by stakeholder group for any individual Delphi item. Second, because item-level responses cannot be associated with demographic categories, we cannot determine whether specific groups (e.g., researchers vs. HCPs) differed in their preferences. We therefore cannot provide the supplemental table requested. We have now added a short clarification to the Methods and Discussion noting that anonymity was intentionally preserved to ensure methodological rigour and that demographic subgroup analyses were not feasible.

"Delphi responses were collected anonymously, and demographic information was stored separately from item-level ratings."

"Because demographic data were not linked to individual item-level ratings, it was not possible to examine voting patterns across stakeholder groups. This reflects the

intentional anonymity of the Delphi design, which aims to minimise social influence and protect the independence of expert judgements."

With regard to the specific point about the referral criteria, this discussion did not emerge from stakeholder-specific voting patterns within the Delphi rounds. Rather, it arose from (a) written qualitative feedback submitted by participants across rounds, (b) the consensus workshop where ambiguity and overlap in item wording were discussed, and (c) iterative consultation with our PPI panel of parents of children with tics, who emphasised that distress, harm, or functional impairment should independently justify referral. The decision to revise the referral criteria therefore reflected a combination of expert deliberation and lived experience input, not demographic voting splits. We have now provided further information about this on page 11 of the manuscript - see also response to point 3 previously discussed.

➢ The following has been added to the Methods *(page 7)*:

"Delphi responses were collected anonymously, and demographic information was stored separately from item-level ratings. This ensured participants could provide honest and independent judgements and prevented item-level analyses by stakeholder group."-level ratings. This ensured participants could provide honest and independent judgements and prevented item-level analyses by stakeholder group

➢ We have also reflected on this in the discussion *(page 16)*:

"Because demographic data were not linked to individual item-level ratings, it was not possible to examine voting patterns across stakeholder groups. This reflects the intentional anonymity of the Delphi design, which aims to minimise social influence and protect the independence of expert judgements."-level ratings, it was not possible to examine voting patterns across stakeholder groups. This reflects the intentional anonymity of the Delphi design, which aims to minimise social influence and protect the independence of expert judgements.

5. Additional Comment: Re: Table 1 - From my view of the paper, part of the table appears to be cut off. Ensure all genders are represented on the table, as currently, I only see Female 69.4% (34), 68.8% (33).

➢ Thank you we have added both genders to Table 1.

VERSION 2 - REVIEW

**Reviewer**	**1**
**Name**	**Ludlow, Amanda**
**Affiliation**	**University of Hertfordshire**
**Date**	**03-Mar-2026**
**COI**	

I thank the authors for the time and consideration given to the queries. They have greatly improved the revised manuscript. The inclusion of further details to Delphi study methodology and recruitment has greatly improved the clarity of the manuscript. I have no further comments.

**Reviewer**	**2**
**Name**	**Young, Bryan**
**Affiliation**	**Douglas Mental Health University Institute**
**Date**	**23-Feb-2026**
**COI**	

Thank you for your thoughtful consideration of my comments. I believe all issues have been adequately addressed, and I particularly appreciated the additions to the discussion and limitations sections of your manuscript regarding some of my earlier points.
